# Does applying the Canadian Cervical Spine rule reduce cervical spine radiography rates in alert patients with blunt trauma to the neck? A retrospective analysis

**DOI:** 10.1186/1471-2342-8-12

**Published:** 2008-06-16

**Authors:** Ulfin Rethnam, Rajam Yesupalan, Giri Gandham

**Affiliations:** 1 Department of Orthopaedics, Glan Clwyd Hospital, Bodelwyddan, UK; 2 Department of Accident & Emergency, Glan Clwyd Hospital, Bodelwyddan, UK

## Abstract

**Background:**

A cautious outlook towards neck injuries has been the norm to avoid missing cervical spine injuries. Consequently there has been an increased use of cervical spine radiography. The Canadian Cervical Spine rule was proposed to reduce unnecessary use of cervical spine radiography in alert and stable patients. Our aim was to see whether applying the Canadian Cervical Spine rule reduced the need for cervical spine radiography without missing significant cervical spine injuries.

**Methods:**

This was a retrospective study conducted in 2 hospitals. 114 alert and stable patients who had cervical spine radiographs for suspected neck injuries were included in the study. Data on patient demographics, high risk & low risk factors as per the Canadian Cervical Spine rule and cervical spine radiography results were collected and analysed.

**Results:**

28 patients were included in the high risk category according to the Canadian Cervical Spine rule. 86 patients fell into the low risk category. If the Canadian Cervical Spine rule was applied, there would have been a significant reduction in cervical spine radiographs as 86/114 patients (75.4%) would not have needed cervical spine radiograph. 2/114 patients who had significant cervical spine injuries would have been identified when the Canadian Cervical Spine rule was applied.

**Conclusion:**

Applying the Canadian Cervical Spine rule for neck injuries in alert and stable patients would have reduced the use of cervical spine radiographs without missing out significant cervical spine injuries. This relates to reduction in radiation exposure to patients and health care costs.

## Background

There has always been a cautious outlook towards suspected cervical spine injuries. A missed significant cervical spine injury can lead to disastrous consequences for the patient. This has lead to clinicians utilising cervical spine radiographs liberally [1]. Is this practice acceptable in the present day?

Studies have shown that the incidence of an acute fracture or spinal injury following a blunt trauma to the neck in a neurologically intact patient who has been mobile after the incident is less than 1% [1]. Cervical spine radiographs in these group of patients are negative for fractures in more than 98% [1]. There is no uniformity in requesting for cervical spine radiographs with variations ranging up to six-fold [2]. This increased use of cervical spine radiographs exposes the patient to unnecessary radiation.

2 decision rules available in the literature to guide the use of cervical spine radiographs are the Canadian Cervical Spine rule (CCS) [1] and the National Emergency X-radiography Utilisation Study (NEXUS) [3]. Comparison between the decision rules revealed the Canadian Cervical Spine rule to be more sensitive and specific [3].

The aim of our study was to assess the reduction in cervical spine radiography rates if the Canadian Cervical spine rule was retrospectively applied to patients who had cervical spine radiographs following blunt trauma to the neck.

## Methods

This was a retrospective study conducted in 2 busy district hospitals over a 3 month period. Ethical approval for the study was deemed not necessary by the National Research Ethics Service, a part of the NHS Research Ethics Committee. All adult patients who had cervical spine radiographs for suspected injury to the neck were included in the study. Children, patients who had cervical spine radiographs for non-trauma related causes, presentation after 48 hours of injury and haemodynamically unstable patients were excluded from the study. All patients were identified from the radiology database and patient records were assessed for high risk & low risk factors based on the Canadian Cervical Spine rule by two independent observers for each hospital. Radiograph reports from the radiologists' were assessed for the presence or absence of significant cervical spine injuries.

Based on the Canadian Cervical Spine rule patients were categorised to having a high risk in sustaining a significant cervical spine injury. High risk patients were those who were > 65 years old, who had sustained a dangerous mechanism of injury or complained of paraesthesia in extremities after the injury. A clinically important cervical spine injury was defined as any fracture, dislocation, or ligamentous instability demonstrated by diagnostic imaging. Clinically unimportant cervical spine injuries generally do not require stabilizing treatment or specialized follow-up. All C-spine injuries were considered clinically important unless the patient was neurologically intact and had 1 of 4 injuries: (1) isolated avulsion fracture of an osteophyte (2) isolated fracture of a transverse process not involving a facet joint (3) isolated fracture of a spinous process not involving the lamina or (4) simple compression fracture involving less than 25% of the vertebral body height [1].

Patients being involved in a simple rear end motor vehicle collision, were sitting in the emergency department, were ambulatory any time after the accident, had delayed onset of neck pain or had absent midline cervical spine tenderness on assessment were categorised as low risk. Records were assessed to check whether patients with any low risk factors had assessment of range of neck movements.

The reduction of cervical spine radiographs if the Canadian Cervical spine rule was applied to these patients was quantified.

## Results

158 patients had cervical spine radiographs during the study period. There were 84 males & 74 females. 44 patients were excluded from the study (Table 1). Of the 114 patients included, 28 patients were categorised as being a high risk of sustaining a significant cervical spine injury either due to their age, or the presence of a dangerous mechanism of injury or the presence of paraesthesia in the extremities (Table 2). 86 patients were categorised as low risk (Table 3). 12 patients had assessment of neck movements. All the 12 patients had >45 degree neck movements to either side. 74 patients did not have assessment of neck movements due to over cautiousness of the attending clinician.

**Table 1:** Patients excluded from study

Records not found	6
> 48 hours since injury	9
Foreign body neck	5
Unstable patients	6
< 16 years	16
Neck pain not related to trauma	2

**Table 2:** Patients with high risk factors

Age >65 years	3
Axial load to head	1
High velocity motor vehicle accident	6
Fall from stairs	3
Motorcycle accident	4
Paraesthesia to extremities	24

**Table 3:** Patients with low risk factors

Simple motor vehicle collision	100
Able to sit in the Emergency department	107
Ambulatory after accident	107
Delayed onset of neck pain	82
Absent midline tenderness	32

Applying the Canadian Cervical spine rule would have reduced the cervical spine radiography rates by 75.4% (86/114) if all the patients in the low risk category had assessment of neck movements and had >45° movement on either side. 2 significant cervical spine injuries were identified from the study group. These injuries were from the high risk group and were identified when the Canadian Cervical spine rule was applied.

## Discussion

In alert & stable patients with suspected neck injuries, the possibility of a significant cervical spine injury is very low [2]. There is a wide variation among clinicians in the use of cervical spine radiography [2]. This is due the potential for serious neurological injury with missed significant cervical spine injuries. There is a fear of medico-legal implications from missed cervical spine injuries [4]. Due to the above mentioned reasons there is an excessive use and reliance on cervical spine radiography even in trauma centres [5].

There is however a very low yield from cervical spine radiographs in alert patients with blunt injury to their neck. The reported incidence of finding a significant injury on the cervical spine series is <3% [6,7]. Although the cervical spine radiograph is a low cost procedure (£14 per 3 film series), its unnecessary excessive use can be cumulative and leads to added health care costs and burden of time to the emergency department staff. There is also an unnecessary radiation exposure to the neck.

There is confusion in the literature regarding the indications for cervical spine radiography with some authors recommending cervical spine radiographs in all patients with injuries above the clavicle [8,9] while others advocate a selective approach [10,11].

The 2 well known clinical decision rules available in the literature are the Canadian Cervical spine rule and the NEXUS (National Emergency X-radiography Utilisation Study) group [3]. These rules provide guidance on cervical spine radiography utilisation in alert & stable patients with suspected neck injury. With the Canadian Cervical spine rule, patients fall into either a high or low risk of sustaining a significant cervical spine injury. To be categorised as high risk, patients either need to be >65 years, have paraesthesia to extremities or should have had a dangerous mechanism of injury. These patients have a cervical spine radiograph. If the patients do not have any risk factors, they are assessed for any low risk factors that allow safe assessment of neck movements. If any low risk factor is identified, a neck movement assessment is done. If the patient is able to actively rotate the neck 45 degrees to either side, radiographs are deferred, if not, cervical spine radiographs are done [1]. The Canadian Cervical spine rule has been found to reduce cervical spine radiography rates and time in hard collar [12].

The NEXUS group advocates no cervical spine radiographs if patients satisfy 5 low risk criteria: absence of midline tenderness, normal level of alertness, no evidence of intoxication, no neurological findings and no painful distracting injuries [3]. A comparison between the Canadian Cervical spine rule and the NEXUS showed the Canadian Cervical spine rule to be more specific and sensitive with less number of missed significant cervical spine injuries [3].

In our study we used the Canadian Cervical spine rule due to its better specificity & sensitivity. All our patients had cervical spine radiography and a significant reduction in radiography rates would have been found if the Canadian Cervical spine rule was applied. The 2 significant cervical spine injuries were identified by the Canadian Cervical spine rule. The results of our study do show the benefits of the Canadian Cervical spine rule in reducing cervical spine radiography rates.

The limitation of our study is that it was retrospective and the sample size was small. Compared to other studies in the literature that quote 25% reduction in radiography rates when applying the Canadian cervical spine rule, we would have had 75% reduction [12]. This could be due to the fact that we have presumed all patients in the low risk category to have had >45° movement on either side. If these patients had neck movements assessed, some of them may have required cervical spine radiographs and therefore made the overall reduction in radiography rates may have been lesser. This is another limitation of our study as there is missing data. Even with this limitation, there would still be a good reduction in radiography rates when applying the rule. Further studies are therefore required to prospectively evaluate the utility of applying the Canadian Cervical spine rule in reducing cervical spine radiography rates.

## Conclusion

The Canadian Cervical spine would have significantly reduced cervical spine radiography rates in alert & stable patients with suspected neck injury. It is a safe and easy clinical decision rule that can be applied in clinical practice. The benefits of using the Canadian Cervical spine rule are reduction of cervical spine radiography rates, reduced health care costs and avoids unnecessary radiation exposure to patients.

## Competing interests

The authors declare that they have no competing interests.

## Authors' contributions

UR, the main author was responsible for conducting the study, acquisition, analysis and interpretation of the data and preparing & revising the manuscript. RY, the co-author was responsible for literature review, data acquisition, interpretation and has approved the final draft. GG, the senior author was responsible for conception and design, supervising the study, proof reading & revising the manuscript and has approved the final draft of the manuscript.

## Pre-publication history

The pre-publication history for this paper can be accessed here:


